# Supplementary material for: A Randomized Controlled Trial Evaluating Outcome Impact of Cilostazol in Patients with Coronary Artery Disease or at a High Risk of Cardiovascular Disease
Source: J Pers Med. 2022 Jun 6;12(6):938. doi: 10.3390/jpm12060938 (PMC9225272; doi:10.3390/jpm12060938)

Supplementary Table S1. Comparisons of the composite endpoints and individual endpoints between participants assigned to cilostazol and placebo (per-protocol analysis).

**Endpoint**	**Cilostazol (*n* = 100)**	**Placebo (*n* = 119)**	**HR (95% CI)**	***p*-Value**
Composite endpoints				
MACE	8 (8.0)	17 (14.3)	0.50 (0.22-1.16)	0.11
MCE	4 (4.0)	18 (15.1)	0.24 (0.08-0.71)	0.01
MACCE	7 (7.0)	20 (16.8)	0.36 (0.15-0.84)	0.02
Individual endpoints				
CV death	1 (1.0)	2 (1.7)	0.60 (0.05-6.58)	0.67
Nonfatal MI	0	4 (3.4)	0.02 (0-47.29)	0.31
Nonfatal stroke	3 (3.0)	4 (3.4)	0.75 (0.17-3.39)	0.71
HHF	2 (2.0)	7 (5.9)	0.31 (0.07-1.51)	0.15
Angina pectoris	4 (4.0)	18 (15.1)	0.24 (0.08-0.71)	0.01
Unplanned coronary revascularization	2 (2.0)	6 (5.0)	0.36 (0.07-1.79)	0.21
Unplanned revascularization	1 (1.0)	7 (5.9)	0.15 (0.02-1.25)	0.08
Major amputation	0	0		
Minor amputation	0	0		

MACE includes CV death, nonfatal MI, nonfatal stroke, HHF, or unplanned coronary revascularization.
MCE includes MI, angina pectoris, or unplanned coronary revascularization.
MACCE includes CV death, nonfatal MI, nonfatal stroke, HHF, major amputation, minor amputation, or unplanned revascularization.
CI, confidence interval; CV, cardiovascular; HHF, hospitalization for heart failure; HR, hazard ratio; MACCE, major adverse cardiovascular and cerebrovascular event; MACE, major adverse cardiovascular event; MCE, major coronary event; MI, myocardial infarction.

Supplementary Table S2. Uni- and multi-variables independently predicting MACCE.

Variable	Uni-variable HR (95% CI)	*p*-Value	Multi-variable HR (95% CI)	*p*-Value
Cilostazol	0.47 (0.23-0.96)	0.04	0.48 (0.23-1.02)	0.055
Age	1.07 (1.02-1.11)	0.002	1.07 (1.02-1.11)	0.003
Peripheral artery disease	1.84 (0.84-4.02)	0.13		
Coronary bypass surgery	1.86 (0.25-13.76)	0.54		
Anemia	2.27 (1.09-4.71)	0.03	-	NS
Aspirin	0.88 (0.44-1.79)	0.73		

CI, confidence interval; HR, hazard ratio; NS, non-significant; MACCE, major adverse cardiovascular and cerebrovascular event.

Supplementary Figure S1.

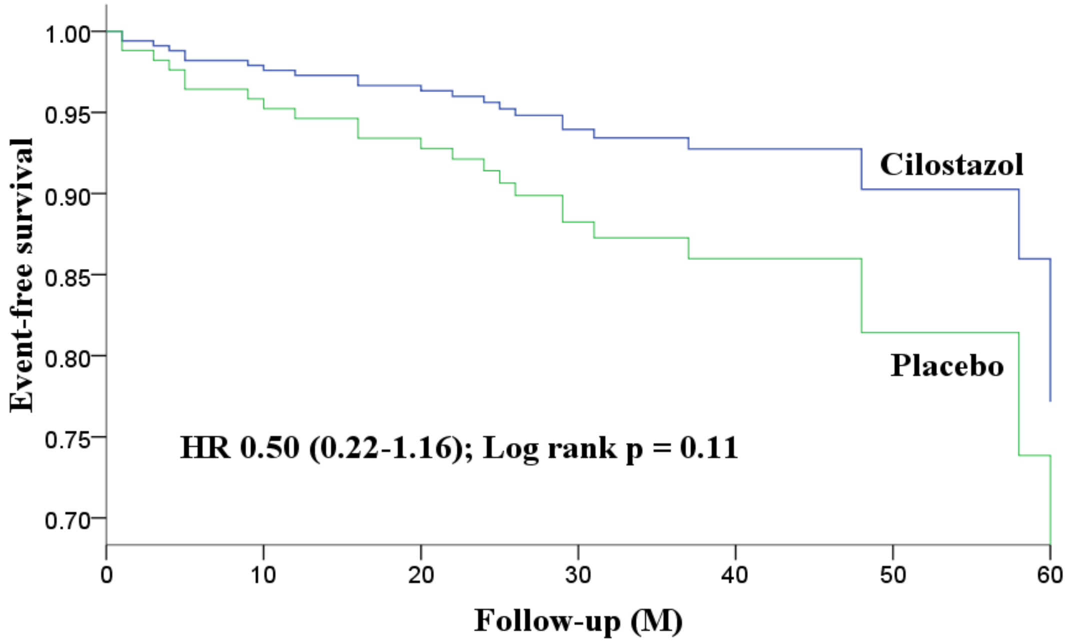

Supplement: Supplementary file 1 [file jpm-12-00938-s001.zip › jpm-1756852-supplementary.pdf]
